# Antimalarial potential of *Moringa oleifera* Lam. (Moringaceae): A review of the ethnomedicinal, pharmacological, toxicological, and phytochemical evidence

**DOI:** 10.1590/1678-9199-JVATITD-2022-0079

**Published:** 2023-05-26

**Authors:** José Jailson Lima Bezerra, Anderson Angel Vieira Pinheiro, Douglas Dourado

**Affiliations:** 1 Graduate Program in Plant Biology, Department of Botany, Federal University of Pernambuco, Recife, PE, Brazil.; 2 Santa Maria University, Cajazeiras, PB, Brazil.; 3 Graduate Program in Biosciences and Biotechnology in Health, Department of Immunology, Aggeu Magalhães-Fiocruz Institute, Recife, PE, Brazil.

**Keywords:** Malaria, Medicinal plants, Flavonoids, Plasmodium, Antiplasmodial

## Abstract

Several regions of the world frequently use the species *Moringa oleifera* Lam. (Moringaceae) in traditional medicine. This situation is even more common in African countries. Many literature reports point to the antimalarial potential of this species, indicating the efficacy of its chemical compounds against malaria-causing parasites of the genus *Plasmodium*. From this perspective, the present study reviews the ethnobotanical, pharmacological, toxicological, and phytochemical (flavonoids) evidence of *M. oleifera*, focusing on the treatment of malaria. Scientific articles were retrieved from Google Scholar, PubMed^®^, ScienceDirect^®^, and SciELO databases. Only articles published between 2002 and 2022 were selected. After applying the inclusion and exclusion criteria, this review used a total of 72 articles. These documents mention a large use of *M. oleifera* for the treatment of malaria in African and Asian countries. The leaves (63%) of this plant are the main parts used in the preparation of herbal medicines. The *in vivo* antimalarial activity of *M. oleifera* was confirmed through several studies using polar and nonpolar extracts, fractions obtained from the extracts, infusion, pellets, and oils obtained from this plant and tested in rodents infected by the following parasites of the genus *Plasmodium*: *P. berghei, P. falciparum, P. yoelii*, and *P. chabaudi*. Extracts obtained from *M. oleifera* showed no toxicity in preclinical tests. A total of 46 flavonoids were identified in the leaves and seeds of *M. oleifera* by different chromatography and mass spectrometry methods. Despite the scarcity of research on the antimalarial potential of compounds isolated from *M. oleifera*, the positive effects against malaria-causing parasites in previous studies are likely to correlate with the flavonoids that occur in this species.

## Background

Human malaria is an infectious disease caused by five species of *Plasmodium* ( *Plasmodium falciparum, Plasmodium vivax, Plasmodium ovale, Plasmodium knowlesi,* and *Plasmodium malariae*) [1]. Its transmission between humans occurs through the bite of female *Anopheles* mosquitoes infected with *Plasmodium* spp. [2]. Among the species of parasites that infect humans, *Plasmodium falciparum* is the main cause of the severe form of the disease, which can lead to death and is responsible for 99.7% of infections in sub-Saharan Africa [3]. Malaria symptoms usually include high fever, headache, muscle aches, vomiting, chills, and fatigue [4]. Despite major advances in the control of this disease, estimates for 2021 suggest a total of about 247 million clinical cases and 619,000 deaths from malaria worldwide [5]. 

Despite the modest reduction in the number of cases over the past 20 years, malaria remains a global health problem [6], even with the production of the Mosquirix^TM^ vaccine, which is not yet widely available [7]. Increasing resistance to drugs currently used in the treatment of this disease is also a serious threat to global malaria control efforts [8]. In this way, the discovery of antimalarial drugs is driven by the need to obtain new therapeutic alternatives to treat infections and save lives in a context of a constantly evolving drug resistance [9]. Herbal medicine has been considered the backbone of malaria treatment for thousands of years. The first antimalarial drug (quinine) was isolated from the bark of a tree of the family Rubiaceae belonging to the genus *Cinchona* [10]. 

Medicinal plants are viable alternatives for the isolation and screening of active phytochemicals that may be responsible for antiplasmodial activity in *in vitro* and *in vivo* assays [11]. In *in vivo* tests, rodents are infected with four different species of *Plasmodium* (*P. berghei, P. chabaudi, P. yoelii*, and *P. vinckei*) and used in research aimed at discovering new antimalarial drugs [12]. Flavonoids apigenin, kaempferol, rutin, and quercetin occur in several plant species and showed promising antimalarial activity in *in vitro* and *in vivo* experiments [13- 18]. In addition, the flavonoids are phytoconstituents with the ability to scavenge free radicals and act as antioxidants [19]. These properties make flavonoids very promising for antimalarial activity, as during malaria infections both the host and the parasites are under severe oxidative stress [20]. In summary, the infected host presents an exacerbated production of free radicals. These free radicals produced in large quantities cause damage to the vascular endothelium, increasing vascular permeability and platelet adhesion, known to be associated with severe cerebral malaria [21, 22]. In this context, flavonoids are promising antioxidants to reverse this clinical condition, deserving a highlight compared to other phytochemicals in the treatment of malaria. 

*Moringa,* the single genus in the family Moringaceae, is one of the most phenotypically varied groups of angiosperms [23, 24]. With only 13 species, *Moringa* occurs in arid regions of Africa, Madagascar, the Arabian Peninsula, and India [23, 25]. The *Moringa*genus has high antioxidant activity mainly due to its high content of flavonoids. Most of the flavonoids present in the genus are in the flavanol and glycoside form [26]. *Moringa oleifera* Lam., popularly known as drumstick and horseradish tree, is native to sub-Himalayan areas of the Indian subcontinent and has been introduced in many tropical countries [25, 27, 28]. Researchers attribute the medicinal, nutritional, and industrial properties of this plant to the constituents that occur in its roots, bark, leaves, flowers, fruits, and seeds [29, 30].

Ethnobotanical and ethnopharmacological surveys carried out in African and Asian countries have reported the use of *M. oleifera* for the treatment of malaria in traditional communities [31- 38]. These ethnomedicinal uses have been confirmed through *in vivo* and *in vitro* assays using different products obtained from the leaves and seeds of *M. oleifera* against several malaria-causing species of *Plasmodium* [39- 44]. It is important to emphasize that the phytochemicals isolated from this species have not yet had their antimalarial activity evaluated in scientific research.

 Considering that malaria still causes several deaths around the world [5] and that the discovery of new antimalarial drugs is of great importance in assisting in the treatment of infections caused by parasites of the genus *Plasmodium* [9], the present study reviews the ethnobotanical, pharmacological, toxicological, and phytochemical (flavonoids) evidence of *M. oleifera*, focusing on the treatment of malaria.

## Methods

### Database search

Scientific articles were retrieved from Google Scholar (https://scholar.google.com.br/), PubMed^®^ (https://pubmed.ncbi.nlm.nih.gov/), ScienceDirect^®^ (https://www.sciencedirect.com/search), and SciELO (https://search.scielo.org/) databases. The keywords used in the searches were: “ *Moringa oleifera* AND ethnobotany AND malaria”, “ *Moringa oleifera* AND medicinal use AND malaria”, “ *Moringa oleifera* AND antimalarial”, “ *Moringa oleifera* AND antiplasmodial”, “ *Moringa oleifera* AND *Plasmodium* AND malaria”, “ *Moringa oleifera* AND toxicity”, “ *Moringa oleifera* AND phytochemistry”, and “ *Moringa oleifera* AND flavonoids”. 

### Inclusion and exclusion criteria

Only scientific articles published between 2002 and 2022 addressing the following information about *M. oleifera* were selected: 1) Ethnomedicinal uses of *M. oleifera* by traditional communities in different regions of the world; 2) *In vitro* and *in vivo* antimalarial activity of extracts, fractions, oils, and other products obtained from *M. oleifera*; 3) Toxicity and biological safety of products obtained from this plant; 4) Flavonoids isolated and identified in *M. oleifera* that have already been reported in the literature. As for the exclusion criteria, review articles, e-books, book chapters, undergraduate theses, Masters’ theses, Ph.D. theses, and works published in technical or scientific events were excluded. 

### Data screening and categorization of information

**Figure 1.**
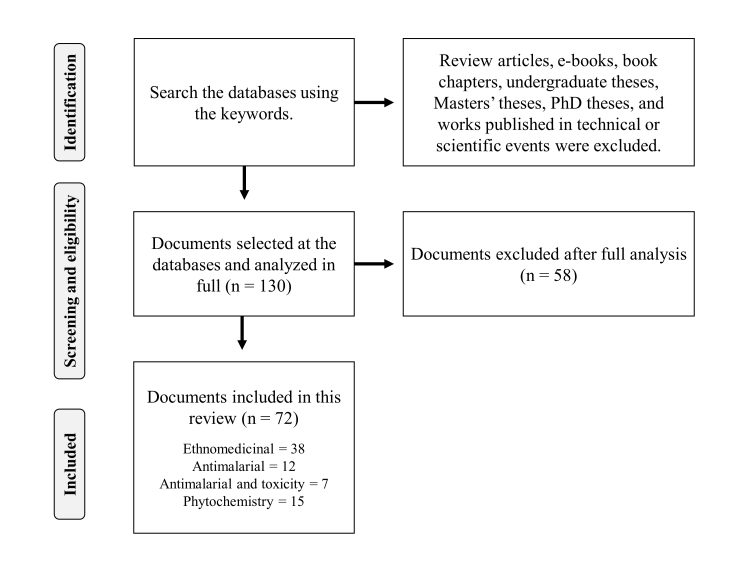
Flow diagram of selection of scientific documents included in this review

A total of 130 scientific articles were selected from the databases (Figure 1). Subsequently, 58 documents that did not meet the criteria of this review were excluded. Finally, the present study considered 72 articles containing data on ethnomedicinal uses, pharmacological activities, and toxicological and phytochemical (flavonoids) investigations of *M. oleifera* focusing on the treatment of malaria (Figure 1, Additional file 1). The results were grouped in tables and represented in graphs when necessary. The general information described in the “Results” section has been categorized by: “Botanical aspects of *Moringa oleifera*”, “Ethnomedicinal uses of *Moringa oleifera* for the treatment of malaria”, “ *In vivo* and *in vitro* antimalarial activity of *Moringa oleifera*”, “Toxicity of *Moringa oleifera*”, and “Flavonoids identified in *Moringa oleifera*”. 

## Results

### Botanical aspects of *Moringa oleifera*

**Figure 2.**
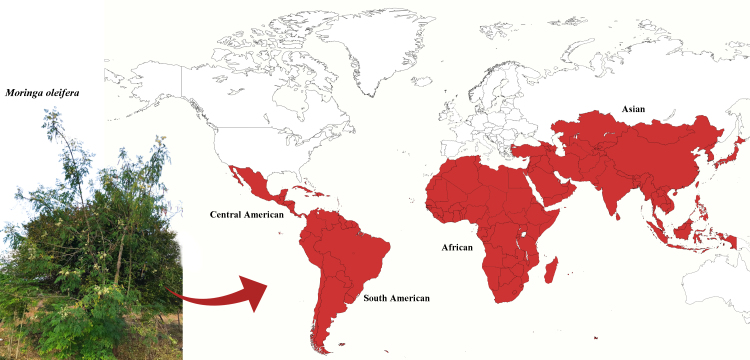
Geographic distribution of *Moringa oleifera*. Map prepared by Bezerra, J.J.L. in MapChart©

*Moringa oleifera* is native to northwest India and adapted to arid and semiarid environments. This plant has gained popularity in certain developing countries due to its medicinal, industrial, and nutritional properties [29]. African, South American, Central American, and Asian countries currently cultivate *M. oleifera* commercially (Figure 2) [45]. This is a medium-sized, fast-growing evergreen tree about 10 to 12 m tall. The bark of mature trees is gray-white while young shoots have a purplish or greenish-white bark [27]. It has a more conventional trunk and fibrous and resistant roots [46]. The fruits are long, woody pods, which when ripe open into three valves, containing trivalve seeds with longitudinal wings. Its pinnate leaves are divided into leaflets arranged on a rachis. The flowers are zygomorphic with five petals, five sepals, five functional stamens, and several staminodes. In addition, the flowers have pedicels and axillary inflorescences [ 47]. 

### Ethnomedicinal uses of *Moringa oleifera* for the treatment of malaria

**Figure 3.**
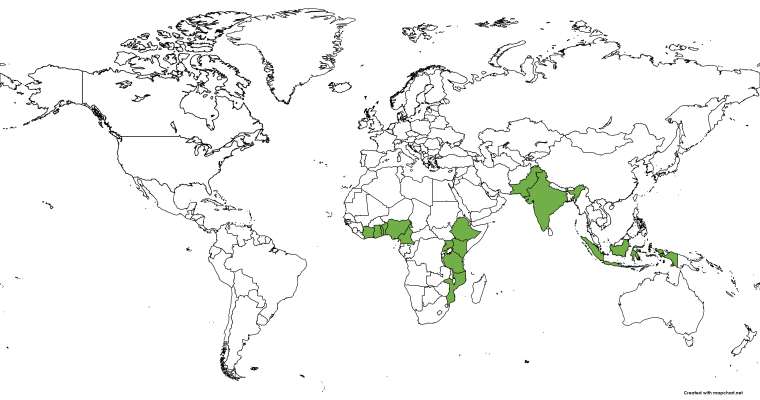
Ethnomedicinal uses of *Moringa oleifera* for the treatment of malaria in African and Asian countries. Map prepared by Bezerra, J.J.L. in MapChart^©^.

The following African countries use *Moringa oleifera* as a traditional medicine for the treatment of malaria: Nigeria, Uganda, Benin, Ghana, Togo, Tanzania, Cameroon, Kenya, Ethiopia, Mozambique, and Cote d’Ivoire. Regarding Asia, ethnobotanical and ethnopharmacological studies have reported the use of this plant for the treatment of malaria in Indonesia, India, and Pakistan (Figure 3). The fact that medicinal indications of *M. oleifera* occur mainly in African countries may correlate directly with the high incidence of cases of this disease in sub-Saharan Africa. In recent years, researchers have carried out several studies on the impact, progression, and control of malaria in this region [48- 50]. 

**Figure 4.**
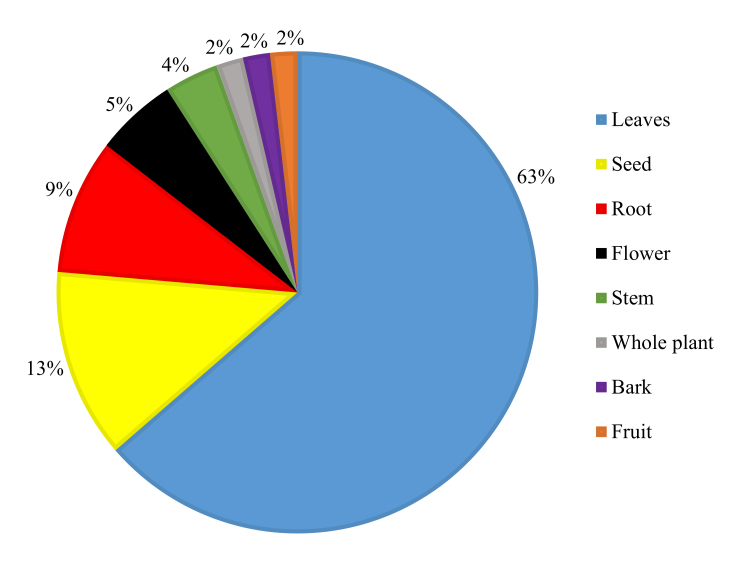
Organs of *Moringa oleifera* used for the treatment of malaria.

**Figure 5.**
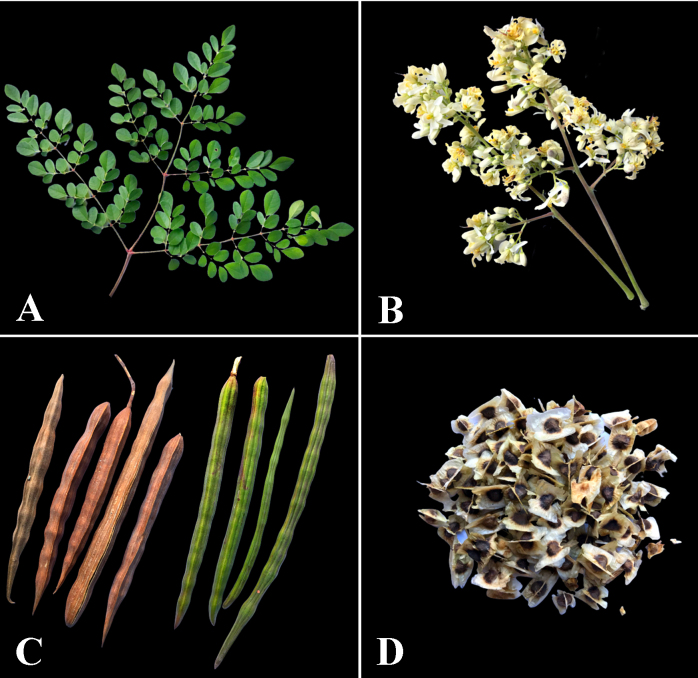
Main organs of *Moringa oleifera* used for the treatment of malaria: **(A)** Leaves, **(B)** Flowers, **(C)** Ripe and unripe fruits, **(D)** Seeds. Photos by: Bezerra, J.J.L.

Traditional communities mainly use *M. oleifera* leaves (63%) for the treatment of malaria. The other most used parts are seeds (13%), roots (9%), flowers (5%), and stems (4%) (Figure 4 and Figure 5). These plant parts are used for the preparation of decoction, maceration, infusion, paste, and cataplasm. Table 1 shows further information on the forms of administration of herbal medicines. The literature often reports a wide use of plant leaves for the treatment of malaria, confirming the findings of this study [51- 53]. 

**Table 1.** Plant organ, preparation mode and ethnomedicinal uses of *Moringa oleifera* for the treatment of malaria.

Plant organ	Preparation mode	Form of administration	Countries	References
Leaves	Maceration, decoction	Oral use	Benin	[ 31]
Leaves	Infusion	1 glass decoction of leaves thrice a day	Pakistan	[ 32]
Leaves	-	-	Ethiopia	[ 33]
Leaves, seed, stem bark	Decoction	-	Nigeria	[ 34]
Root	Paste, cataplasm	-	Indonesia	[ 35]
Leaves	*In natura*	Chew the leaves seven times a day	Uganda	[ 36]
Leaves, bark	Decoction, maceration	-	Nigeria	[ 37]
Bark	-	-	India	[ 38]
Leaves	Boil in water as infusion	Drink the extract	Nigeria	[ 54]
Leaves	Decoction	Oral use, bath	Nigeria	[ 55]
Leaves	-	-	Nigeria	[ 56]
Leaves	Decoction, maceration	-	Nigeria	[ 57]
Leaves	-	-	Nigeria	[ 58]
Leaves	Decoction, maceration	-	Nigeria	[ 59]
Leaves	Decoction	Boil leaves for 20 minutes and consume half cup morning and evening	Nigeria	[ 60]
Leaves	Ground leaves + little water	Drink regularly 3 small glasses/day until you are healed	Nigeria	[ 61]
Leaves	-	-	Uganda	[ 62]
Leaves	Decoction	-	Uganda	[ 63]
Leaves	Decoction, maceration	Oral use	Benin	[ 64]
Leaves	Ground leaves + little water	Drink regularly 3 small glasses/day until well	Benin	[ 65]
Leaves	Aqueous boiling	-	Ghana	[ 66]
Leaves	Decoction	Boil and drink one cupful of decoction thrice daily for 3-5 days or mash leaves in water and drink thrice daily	Ghana	[ 67]
Leaves, seed	Leaves boiled in strained corn ( *Zea mays* L.) dough liquid	-	Ghana	[ 68]
Leaves	Maceration	Body bath	Togo	[ 95]
Leaves	-	-	Benin	[ 96]
Leaves, flower	Maceration	One spoon twice daily	Nigeria	[ 97]
Leaves	Decoction. Dry powder used to make warm infusion	-	Tanzania	[ 98]
Leaves, root	Decoction. Boil handful of fresh leaves in a cup of water.	Drink one glass 3 times a day for adults. For children, give 1 tsp 3 times a day. Roots chewed raw or boiled and drunk 2 times a day. Pick and chew handful of fresh leaves 2-3 times a day for 3-4 days	Uganda	[ 99]
Leaves, seed	-	-	Cameroon	[ 100]
Flower, leaves, seed, root	Infusion	Chewed, boiled and infusion taken orally	Kenya	[ 101]
Leaves, seed	Cold maceration	-	Nigeria	[ 102]
Fruit	-	-	Togo	[ 103]
Leaves, flower	Decoction	Drinking, eating	Nigeria	[ 104]
Leaves, roots	Decoction	Chewed raw	Uganda	[ 105]
Leaves, seed, whole plant	Decoction, infusion	-	Nigeria	[ 106]
Root, stem, leaves	-	-	Mozambique	[ 107]
Leaves, seed	-	-	Nigeria	[ 108]
Leaves	Decoction, infusion	Oral use	Cote d’Ivoire	[ 109]

Ethnobotanical and ethnopharmacological surveys carried out in Nigeria have highlighted the constant use of *M. oleifera* leaves for the treatment of malaria by traditional communities in the country [54- 61]. Other African countries that also stood out in the use of *M. oleifera* leaves were Uganda [36, 62, 63], Benin [31, 64, 65], and Ghana [66- 68]. Scientific research shows that extracts from the leaves of this plant had *in vivo* antimalarial activity [69- 72], confirming its use in traditional medicine.

### *In vivo* and *in vitro* antimalarial activity of *Moringa oleifera*

Several studies have reported the *in vivo* and *in vitro* antimalarial activity of *M. oleifera* (Table 2). Researchers mostly used *in vivo* methods to evaluate the potential of polar and nonpolar extracts, fractions obtained from extracts, infusions, pellets, and oils obtained from this plant and tested in rodents infected by the following parasites of the genus *Plasmodium*: *P. berghei, P. falciparum, P. yoelii*, and *P. chabaudi*. Leaves were the most used parts to obtain the evaluated products. Regarding *in vitro* tests, only two studies reported the potential of *M. oleifera* against the parasite *P. falciparum*. This parasite infects humans and causes the most severe form of malaria [40, 73].

**Table 2.** Plant organ and phytoproducts concentration of phytoproducts *Moringa oleifera* for i *n vivo* and *in vitro* antimalarial activity.

Plant organ	Product	Concentration or dose	Strain	Method	References
Leaves	Acetone extract	200, 400 and 600 mg/kg	*Plasmodium berghei*	*In vivo*	[ 39]
Leaves	Hexane extract, methanol extract, aqueous extract	6.25, 12.5, 25 and 50 µg/mL	*Plasmodium falciparum* (3D7)	*In vitro*	[ 40]
Leaves	Aqueous extract, ethanol extract	100, 200, 400 and 800 mg/kg	*Plasmodium berghei*	*In vivo*	[ 41]
Seed	Ethanol extract	200, 300 and 500 mg/kg	*Plasmodium berghei*	*In vivo*	[ 42]
Leaves	Pellets	30 and 60 mg/mouse	*Plasmodium chabaudi*	*In vivo*	[ 43]
Seed	Oil	200, 400 and 800 mg/kg	*Plasmodium berghei* (NK65)	*In vivo*	[ 44]
Leaves	Aqueous extract	250, 500, 750 and 1000 mg/kg	*Plasmodium berghei* (NK65)	*In vivo*	[ 69]
Leaves	Aqueous extract	100, 500 and 1000 mg/kg	*Plasmodium berghei* (ANKA)	*In vivo*	[ 70]
Leaves	Aqueous extract	500, 1000 and 2000 mg/kg	*Plasmodium berghei* (ANKA)	*In vivo*	[ 71]
Leaves	Aqueous extract	100, 500 and 1000 mg/kg	*Plasmodium berghei* (ANKA)	*In vivo*	[ 72]
Flowers, leaves, stems	Lipophilic extract, methanol extract	6 and 25 µg/mL	*Plasmodium falciparum*	*In vitro*	[ 73]
Leaves	Methanol extract and its fractions	50 and 100 mg/kg	*Plasmodium berghei* (NK65)	*In vivo*	[ 74]
Leaves	Aqueous extract	100, 1000 and 2000 mg/kg	*Plasmodium berghei* (ANKA)	*In vivo*	[ 75]
Leaves	*n*-Hexane extract, ethanol extract	50, 100 and 200 mg/kg	*Plasmodium berghei*	*In vivo*	[ 76]
Flowers, leaves	Methanol extract	125, 250, 500 and 1000 mg/kg	*Plasmodium yoelii* (N-67)	*In vivo*	[ 77]
Seed	*n*-Hexane extract, ethanol extract	50, 100 and 200 mL/kg	*Plasmodium berghei*	*In vivo*	[ 110]
Leaves	Bioactive fraction	100, 150 and 200 mg/kg	*Plasmodium berghei* Q (N1923)	*In vivo*	[ 111]
Leaves	Aqueous extract	150 mg/kg	*Plasmodium yoelii*	*In vivo*	[ 112]
Leaves	Infusion	100, 200 and 400 mg/kg	*Plasmodium berghei* (ANKA)	*In vivo*	[ 113]

According to Orman et al. [69], the parasitic suppression of the aqueous extract of *M. oleifera* leaves was not entirely dose-dependent in mice. This is because the two lowest doses, 250 mg/kg (69.31% of suppression) and 500 mg/kg (77.26% of suppression), exhibited better suppression of *P. berghei* (NK65) than the two highest doses, 750 mg/kg (25.28% of suppression) and 1000 mg/kg (7.12% of suppression). In turn, Ogundapo et al. [74] observed in their *in vivo* antimalarial studies that the methanolic extract of *M. oleifera* leaves (50 and 100 mg/kg) was able to suppress 42.37 and 55.30 %, respectively, the parasitemia induced by *P. berghei*. Somsak et al. [71] reported that the aqueous extract of *M. oleifera* leaves at doses of 500, 1000, and 2000 mg/kg showed antimalarial activity of 35, 40, and 50%, respectively, against *P. berghei*.

Dondee et al. [70] observed that the aqueous extract of *M. oleifera* leaves significantly inhibited parasitemia in mice infected with *P. berghei* in a dose-dependent manner. Percent inhibitions of 42.86, 71.43, and 85.71% occurred at doses of 100, 500, and 1000 mg/kg of the extract, respectively. Dondee et al. [75] also reported results similar to these, but evaluated doses 100, 1000, and 2000 mg/kg. Despite the concentration-dependent behavior, it can be inferred that due to the low variation between the doses of 1000 and 2000 mg/kg, in the suppression of the parasitemia it is already close to a maximum concentration tending to a constant. At the dose of 200 mg/kg, ethanolic and n-hexane extracts from *M. oleifera* leaves revealed 98.3 and 100% (suppression) of total parasitemia in mice infected with *P. berghei*, respectively [76]. Mulisa et al. [39] reported that the acetone extract from *M. oleifera* leaves at doses of 200, 400, and 600 mg/kg suppressed *P. berghei* parasitemia by 31.1, 55.9, and 77.0%, respectively.

The dose of 800 mg/kg of aqueous and ethanolic extracts of *M. oleifera* leaves suppressed *P. berghei* infection in mice by 99.48 and 97.75%, respectively [41]. According to Obediah and Obi [42], doses of 200 mg/kg (68.93% of suppression), 300 mg/kg (72.56% of suppression), and 500 mg/kg (67.01% of suppression) of the ethanol extract of *M. oleifera* seeds showed good chemosuppression of *P. berghei* multiplication in relation to the negative control. According to Shrivastava et al. [77], extracts of *M. oleifera* flowers and leaves showed dose-dependent suppression in mice infected with the parasite *P. yoelii* (N-67). At the lowest dose (125 mg/kg), the flower and leaf extracts suppressed infection by 40.74 and 31.85 %, respectively, after four days of experiment [77].

In an *in vitro* experiment, Daskum et al. [40] tested different extracts of *M. oleifera* leaves against the *P. falciparum* strain 3D7. According to these authors, although some extracts were more potent than others, all were biologically active with the following IC_50_ values: hexane extract IC_50_ = 3.36 µg/mL; methanolic extract IC_50_ = 3.44 µg/mL; aqueous extract IC_50_ = 4.09 µg/mL. It is important to highlight that the most severe form of malaria and the mortality rate in humans often correlate with infections caused by *P. falciparum* [78- 80]. Thus, studies focusing on the evaluation of new drugs against this specific parasite are of great importance for public health.

### Toxicity of *Moringa oleifera*

Researchers evaluated the toxicity of different products obtained from *M. oleifera* in experimental rodent models [ 39, 42, 70, 71, 75, 77]. These studies regarded the extracts obtained from this species as biologically safe since the evaluated animals did not present relevant behavioral or physiological changes during the acute and subacute toxicity experiments. However, despite the extracts being considered safe, a recent study by Abdulahi et al. [44] reported that precautions should be taken when administering *M. oleifera* seed oil at a dose greater than 200 mg/kg, as this product may be mildly toxic.

According to Somsak et al. [71], the aqueous extract of *M. oleifera* leaves orally administered in a single dose of up to 4000 mg/kg showed no visible signs of toxicity (paw licking, salivation, stretching, urination, lacrimation, hair erection, and reduction in feeding activity) in mice. Additionally, no mortality occurred within the observation period of 30 days. Dondee et al. [70] observed similar results, reporting that the aqueous extract of *M. oleifera* leaves administered orally at a dose of up to 4000 mg/kg also did not cause mortality in mice over the seven days of observation. When orally administering the single dose of 2000 mg/kg of the aqueous extract of *M. oleifera* leaves to mice, Dondee et al. [75] reported the absence of lethal effect in the animals up to one week after the experiment. However, at the dose of 4000 mg/kg, these authors observed tremors and drowsy activities after 24 hours of extract administration.

In a study carried out by Mulisa et al. [39], the acetone extract of *M. oleifera* leaves did not result in animal death at the dose of 2000 mg/kg. This implies that the lethal dose (LD_50_) of the extract was greater than 2000 mg/kg. Moreover, Obediah and Obi [42] reported that the ethanol extract of *M. oleifera* seeds was not considered toxic at the highest dose of 911 mg/kg administered to albino rats. According to Shrivastava et al. [77], the results of the subacute toxicity evaluation indicated that both methanolic extracts (flower and leaves) were not considered toxic to mice, even at the highest dose level (3000 mg/kg), in the first 24 h, as well as in the following 14 days of the experiment.

### Flavonoids identified in *Moringa oleifera*

**Figure 6.**
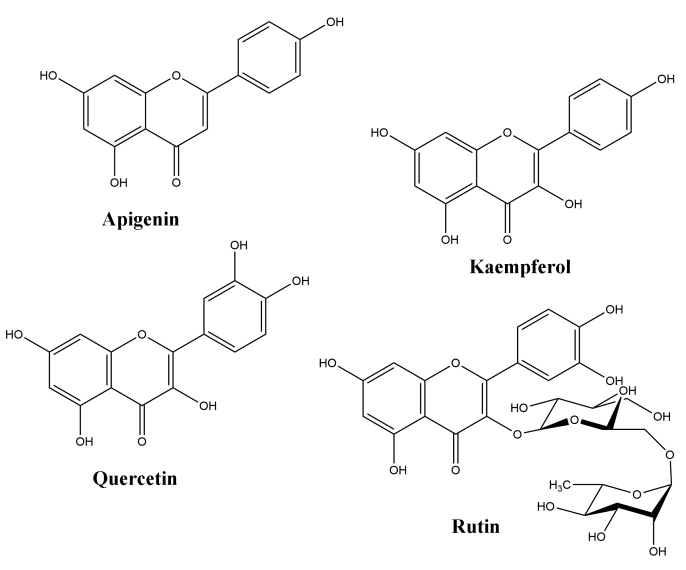
Flavonoids with antimalarial potential.

By using different chromatography and mass spectrometry methods, researchers identified a total of 46 flavonoids in *M. oleifera* leaves and seeds (Table 3). Some examples of these flavonoids are apigenin [81, 82], kaempferol [81, 82], rutin [ 83, 84], and quercetin [84- 85] (Figure 6). To date, however, the literature does not mention the antimalarial potential of phytochemicals isolated from this plant. Several other studies have reported the promising antimalarial potential of apigenin, kaempferol, rutin, and quercetin in *in vitro* and *in vivo* experiments [13- 18]. The antimalarial activity of *M. oleifera* may thus correlate directly with these flavonoids. Flavonoids are usually well known to show effects against malaria-causing parasites [86- 89].

**Table 3:** Plant organ, identification methods, and flavonoids present in *Moringa oleifera*.

Compound	Plant organ	Identification method	References
1) Quercetin 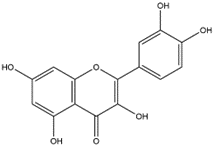	Seed, leaves	HPLC, UHPLC-ESI-QTOF-MS/MS, NMR, ESIMS, HPLC-Q-TOF-MS/MS, HPLC-DAD, LC-MS	[ 81, 82, 84, 114- 118]
2) Epicatechin 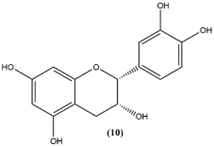	Seed, leaves	HPLC, LC-MS	[ 82, 114]
3) Catechin 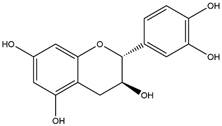	Seed, Leaves	HPLC	[ 85, 114]
4) Isoquercetin 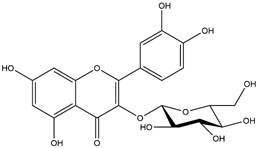	Leaves	HPLC	[ 119]
5) Kaempferol acetyl dihexose 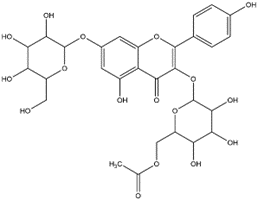	Leaves	UHPLC-qTOF-MS	[ 120]
6) Quercetin acetyl dihexose 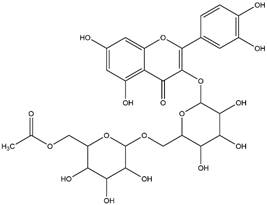	Leaves	UHPLC-qTOF-MS	[ 120]
7) Quercetin hexose 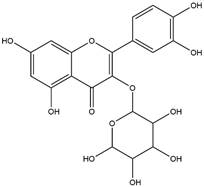	Leaves	UHPLC-qTOF-MS	[ 120]
8) Quercetin hydroxy-methylglutaroyl hexose 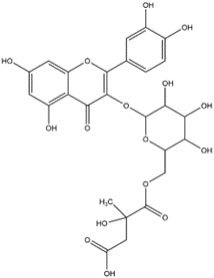	Leaves	UHPLC-qTOF-MS, UHPLC-ESI-QTOF-MS/MS	[ 116, 120]
9) Quercetin acetyl hexose 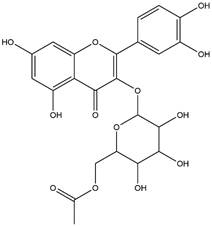	Leaves	UHPLC-qTOF-MS	[ 120]
10) Kaempferol hexose 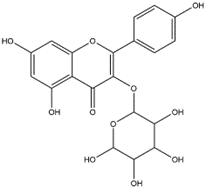	Leaves	UHPLC-qTOF-MS	[ 120]
11) Isorhamnetin hexose 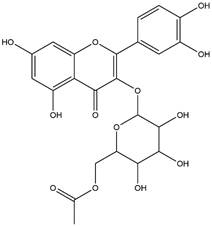	Leaves	UHPLC-qTOF-MS	[ 120]
12) Quercetin malonyl hexose 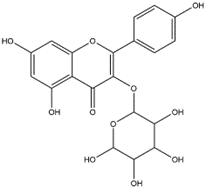	Leaves	UHPLC-qTOF-MS	[ 120]
13) Kaempferol hydroxy-methylglutaroyl hexose 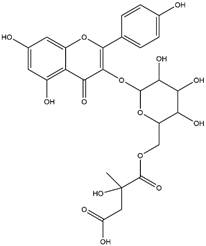	Leaves	UHPLC-qTOF-MS	[ 120]
14) Kaempferol acetyl hexose 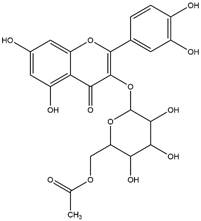	Leaves	UHPLC-qTOF-MS	[ 120]
15) Kaempferol malonyl hexose 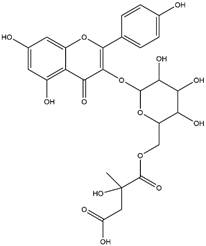	Leaves	UHPLC-qTOF-MS	[ 120]
16) Isorhamnetin hydroxy-methylglutaroyl hexose 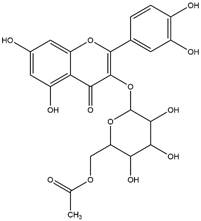	Leaves	UHPLC-qTOF-MS	[ 120]
17) Isorhamnetin acetyl hexose 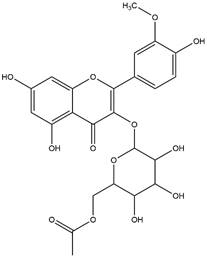	Leaves	UHPLC-qTOF-MS	[ 120]
18) Luteolin-6-C-glucoside 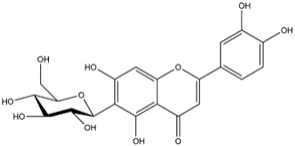	Seed	LC-MS	[ 121]
19) Hesperidin 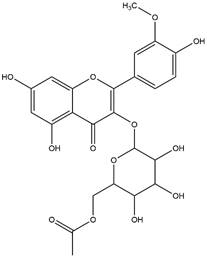	Seed	LC-MS	[ 121]
20) Kaempferol 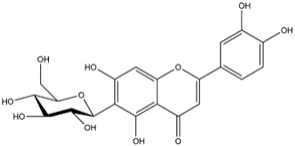	Leaves	UHPLC-ESI-QTOF-MS/MS, NMR, ESIMS, HPLC-Q-TOF-MS/MS, HPLC-DAD, LC-MS	[ 81, 82, 84, 115- 118]
21) Myricetin 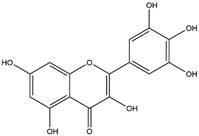	Leaves	HPLC	[ 115]
22) Rutin 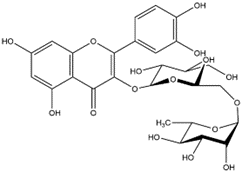	Leaves	HPLC-ESI-MS, HPLC-Q-TOF-MS/MS, LC-MS, HPLC-DAD, UHPLC-Q-Exactive-MS/MS	[ 81- 85, 122, 123]
23) Vitexin 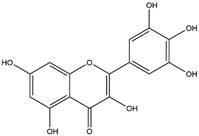	Leaves	UHPLC-ESI-QTOF-MS/MS, UHPLC-Q-Exactive-MS/MS	[ 116, 123]
24) Astragalin 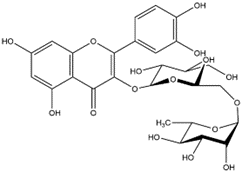	Leaves	UHPLC-ESI-QTOF-MS/MS, UHPLC-Q-Exactive-MS/MS	[ 116, 123]
25) Quercetin-3-glucoside 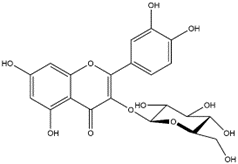	Leaves	HPLC-ESI-MS, HPLC-DAD	[ 83, 122]
26) Kaempferol-3- *O-*glucoside 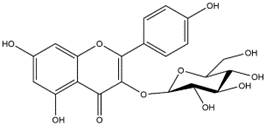	Leaves	HPLC-ESI-MS, HPLC-DAD	[ 83, 122]
27) Quercetin-3-acetyl-glucoside 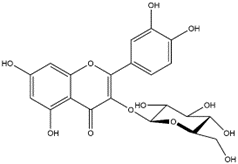	Leaves	HPLC-ESI-MS, UHPLC-ESI-QTOF-MS/MS, HPLC-DAD	[ 83, 116, 122]
28) Quercetin 3- *β*-D-glucoside 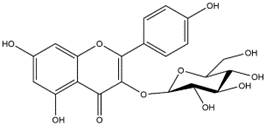	Leaves	UHPLC-ESI-QTOF-MS/MS	[ 116]
29) Isovitexin 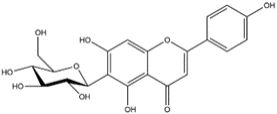	Leaves	UHPLC-ESI-QTOF-MS/MS	[ 116]
30) Kaempferol-3- *O*-rutinoside 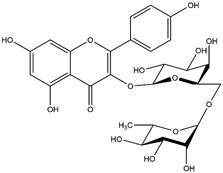	Leaves	HPLC-Q-TOF-MS/MS, HPLC-DAD	[ 81, 84]
31) Quercetin 3- *O*-galactoside 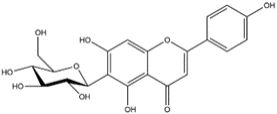	Leaves	HPLC-DAD	[ 118]
32) Quercetin 3- *O*-rhamnoside 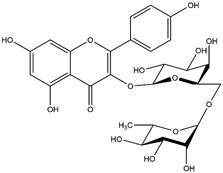	Leaves	HPLC-DAD	[ 118]
33) Kaempferol 3- *O*-galactoside 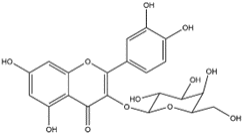	Leaves	HPLC-DAD	[ 118]
34) Kaempferol 3- *O*-glucoside 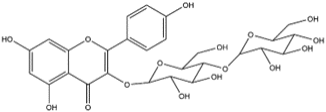	Leaves	HPLC-DAD	[ 118]
35) Hyperoside 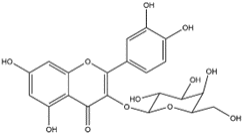	Leaves	HPLC-Q-TOF-MS/MS, HPLC-DAD	[ 81, 84]
36) (−)-Epigallocatechin 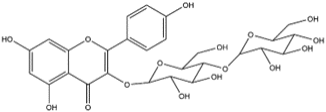	Leaves	HPLC-Q-TOF-MS/MS	[ 81]
37) Vicenin-2 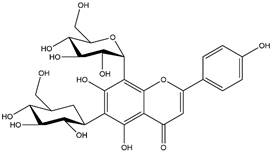	Leaves	UHPLC-qTOF-MS, HPLC-Q-TOF-MS/MS	[ 81, 120]
38) Orientin 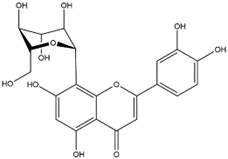	Leaves	HPLC-Q-TOF-MS/MS	[ 81]
39) Apigenin 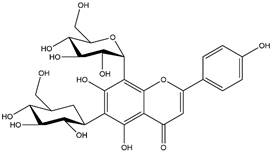	Leaves	HPLC-Q-TOF-MS/MS, LC-MS, HPLC-DAD	[ 81, 82, 84]
40) Quercitrin 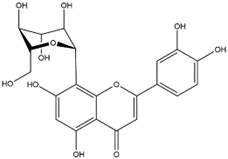	Leaves	LC-MS	[ 82]
41) Naringin 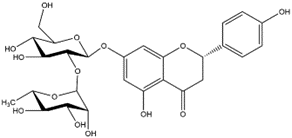	Leaves	LC-MS	[ 82]
42) Naringenin 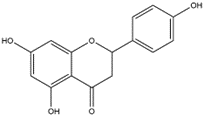	Leaves	LC-MS	[ 82]
43) Luteolin 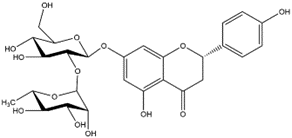	Leaves	LC-MS	[ 82]
44) Cirsilineol 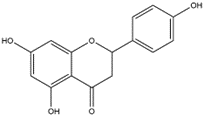	Leaves	LC-MS	[ 82]
45) Acacetin 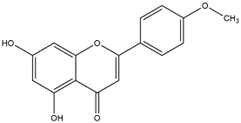	Leaves	LC-MS	[ 82]
46) Isoquercitrin 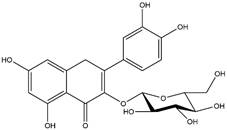	Leaves	HPLC-DAD, UHPLC-Q-Exactive-MS/MS	[ 123]

ESIMS: Electrospray ionization mass spectrometry; HPLC: High performance liquid chromatography; HPLC-DAD: High performance liquid chromatography - diode array detector; HPLC-ESI-MS: High-performance liquid chromatography coupled to electrospray ionization tandem mass spectrometry; HPLC-Q-TOF-MS/MS: High performance liquid chromatography-quadrupole time-of-flight tandem mass spectrometry; LC-MS: Liquid chromatography - mass spectrometry; NMR: Nuclear magnetic resonance; UHPLC-qTOF-MS: Ultra-high performance liquid chromatography-quadrupole time-of-flight mass spectrometry; UHPLC-ESI-QTOF-MS/MS: Ultra-high pressure liquid chromatography accurate mass quadrupole time-of-flight mass spectrometry with electrospray ionization; UHPLC-Q-Exactive-MS/MS: Ultra--high performance liquid chromatography-quadrupole-electrostatic field Orbitrap mass spectrometry.

## Discussion

Despite the many reports of the use of *M. oleifera* in traditional medicine for the treatment of malaria in several countries and the evaluation of its extracts for their antimalarial potential in *in vitro* and *in vivo* experiments, this study identified some research gaps. Initially, it is important to note the absence of studies on the antimalarial activity of phytochemicals isolated from this plant. This fact makes it difficult, for example, to elucidate the mechanisms of action of compounds isolated from *M. oleifera* against malaria-causing parasites of the genus *Plasmodium*. Pan et al. [90] report the isolation of several antimalarial compounds from plants during the last decade, with many of these compounds showing significant *in vitro* activity against *P. falciparum*. These studies are essential for the discovery of new antimalarial drugs.

When evaluating the performance of an extract instead of isolated molecules, you have a phytocomplex, which can have synergistic compounds, as well as interfering compounds (PAINs). For the use of these extracts, standardization is recommended based on biomarkers that can be correlated with pharmacological activity, such as the flavonoids present in this species.

In addition to the metabolic composition, recent studies have pointed to a potential role for the microRNA of this species in the production of key molecules capable of justifying the bioactivity of this plant [91, 92]. In this perspective, studies of *in vitro* cell culture and callus of *M. oleifera* have already been carried out for the massive production of secondary metabolites and microRNA that present biological properties [93]. These studies bring perspectives for optimizing and obtaining pharmacologically active metabolites.

Furthermore, the present study did not find reports of randomized clinical trials of products obtained from *M. oleifera* that can be used in the treatment of malaria. Randomized clinical trials are needed before herbal remedies can be recommended on a large scale. As these studies are expensive and time-consuming, it is important to prioritize new drugs for clinical investigation according to existing data from sociological, ethnobotanical, pharmacological studies, and preliminary clinical observations [94]. Moreover, the observed *in vitro* studies were not carried out with resistant strains of Plasmodium, providing yet another gap in this study. 

Therefore, considering the widespread use of *M. oleifera* by traditional communities for the treatment of malaria and the vast scientific evidence on its antimalarial potential in preclinical studies, it is important to carry out *in vitro* assays with resistant strains and clinical trials to ensure the effective and safe use of products obtained from this plant in humans.

## Conclusion

Africans and Asians make large use of *Moringa oleifera* for the treatment of malaria. The leaves of this plant are the main parts used in the preparation of herbal medicines. The *in vivo* antimalarial activity of *M. oleifera* was confirmed through several studies using polar and nonpolar extracts, fractions obtained from the extracts, infusion, pellets, and oils obtained from this plant and tested in rodents infected by the following parasites of the *Plasmodium* genus: *P. berghei, P. falciparum, P. yoelii*, and *P. chabaudi*. Extracts obtained from *M. oleifera* showed no toxicity in preclinical tests. By using different chromatography and mass spectrometry methods, researchers identified a total of 46 flavonoids in *M. oleifera* leaves and seeds. Despite the scarcity of studies on the antimalarial potential of compounds isolated from *M. oleifera*, the positive effects against malaria-causing parasites observed in previous studies are likely to correlate with the flavonoids that occur in this species.

## Supplementary material

The following online material is available for this article:

Additional file 1. Characterization of the articles selected in the databases and included in the review (n = 72).
