# Supplementary material for: Antimalarial potential of Moringa oleifera Lam. (Moringaceae): A review of the ethnomedicinal, pharmacological, toxicological, and phytochemical evidence
Source: J Venom Anim Toxins Incl Trop Dis. 2023 May 26;29:e20220079. doi: 10.1590/1678-9199-JVATITD-2022-0079 (PMC10231345; doi:10.1590/1678-9199-JVATITD-2022-0079)
Supplement: Additional file 1. [file 1678-9199-jvatitd-29-e20220079-s1.pdf]

J Venom Anim Toxins incl Trop Dis
https://doi.org/10.1590/1678-9199-JVATITD-2022-0079

Supplementary Material to "Antimalarial potential of *Moringa oleifera* Lam. (Moringaceae): A review of the ethnomedicinal, pharmacological, toxicological, and phytochemical evidence"

Additional file 1. Characterization of the articles selected in the databases and included in the review (n = 72).

	**Ethnomedicinal (n = 38)**	
**Database**	**Title**	**Authors, year**
Google Scholar	Ethnobotanical assessment of *Moringa oleifera* Lam. in southern Benin (West Africa) [1]	Agoyi et al., 2014
	Ethnobotanical study of medicinal plants used for the treatment of malaria in Nupeland, North Central Nigeria [2]	Nda-Umar et al., 2014
	Herbal medicinal treatment of malaria in Aliero local government area, Kebbi, Nigeria [3]	Singh and Singh, 2014
	Ethnobotanical survey and *in vitro* antiplasmodial activity of medicinal plants used to treat malaria in Kagera and Lindi regions, Tanzania [4]	Nondo et al., 2015

Ethnobotanical survey of plants used in the treatment of malaria in the Sekyere Central District of Ashanti Region of Ghana [5]	Yaw et al., 2015
Medicinal plants used in the treatment and prevention of malaria in Cegere Sub-County, Northern Uganda [6]	Anywar et al., 2016
Ethnobotanical survey of medicinal plants used in malaria management in South Benin [7]	Lagnika et al., 2016
Ethnobotanic survey of medicinal plants used for malaria therapy in western Cameroon [8]	Olivier et al., 2016
Ethnobotanical survey of mangrove plant species used as medicine from Ouidah to Grand-Popo Districts, Southern Benin [9]	Dossou-Yovo et al., 2017
Ethnobotanical survey of plants used in the treatment of typhoid and its complication(s) in esan north east local Government Area, Uromi, Edo State [10]	Igberaese and Ogbole, 2018
Ethnobotanical Survey of Medicinal Plants in Mwala Sub-County, Machakos County, Kenya [11]	Mutwiwa et al., 2018
An ethnobotanical survey of medicinal plants used in the preparation of “Atikédi”: Local alcoholic beverages commonly consumed in Lomé Togo [12]	Yaovi-Gameli et al., 2018

Ethno botanical survey of medicinal flora used for the treatment of malaria in Madobi Town, Kano State Nigeria [13]	Mukhtar et al., 2019
Management of malaria: an account by the indigenous people of Kashere and its environs, Gombe State, Nigeria [14]	Gani et al., 2019
An ethnobotanical study of plants used for the treatment of malaria in Budondo sub-county, Eastern Uganda [15]	Malinga et al., 2020
A review for selecting medicinal plants commonly used for malaria in Uganda [16]	Ajayi et al., 2020a
Urban forestry and ethno medicine: the meeting point in malaria control in Ijesa Region, Nigeria [17]	Cole and Kayode, 2020
Ethno-botanical survey and phytochemical analysis of *Moringa oleifera* in mubi local government of Adamawa state [18]	Zakawa et al., 2020
Survey of medicinal plants used for the treatment of malaria in Kaduna State, Nigeria [19]	Dogara et al., 2021
Assessment of indigenous knowledge on medicinal plants used in the management of malaria in Kafin Hausa, northwestern Nigeria [20]	Zakariya et al., 2021
Levantamento etnobotânico de plantas medicinais utilizadas pela comunidade de Nacuale, no Parque Nacional das Quirimbas, Moçambique [21]	Muchaia and Nanvonamuquitxo, 2021

	Comprehensive list of anti-malarial plants used by different communities of Assam and Arunachal Pradesh, India [22]	Saikia et al., 2021
	Antimalarial and antianemic medicinal plants used by traditional medicine practitioners and the populations of the Korhogo 1 health district (Poro Region, Ivory Coast) [23]	Kroa et al., 2022
ScienceDirect	Herbal medicines used in the treatment of malaria in Budiope county, Uganda [24]	Tabuti, 2008
	Ethnopharmacological use of herbal remedies for the treatment of malaria in the Dangme West District of Ghana [25]	Asase et al., 2010
	An ethnobotanical study of antimalarial plants in Togo Maritime Region [26]	Koudouvo et al., 2011
	Local knowledge, use pattern and geographical distribution of *Moringa oleifera* Lam. (Moringaceae) in Nigeria [27]	Popoola and Obembe, 2013
	Ethnobotanical study of medicinal plants used for the treatment of malaria in plateau of Allada, Benin (West Africa) [28]	Yetein et al., 2013
	Ethnopharmacological survey of herbal treatment of malaria in Lagos, Southwest Nigeria [29]	Ishola et al., 2014
	Ethnomedicinal survey of plants used in the treatment of malaria in southern Nigeria [30]	Iyamah and Idu, 2015

	Medicinal plants and finished marketed herbal products used in the treatment of malaria in the Ashanti region, Ghana [31]	Komlaga et al., 2015
	Ethnomedicinal uses of plants for the treatment of malaria in Soon Valley, Khushab, Pakistan [32]	Shah and Rahim, 2017
	Treatment of malaria and related symptoms using traditional herbal medicine in Ethiopia [33]	Suleman et al., 2017
	Medicinal plants used by the people of Nsukka Local Government Area, south-eastern Nigeria for the treatment of malaria: An ethnobotanical survey [34]	Odoh et al., 2018
	Ethnobotanical survey of the plants used for the management of malaria in Ondo State, Nigeria [35]	Oyeyemi et al., 2019
	Ethnomedicine of Tetun ethnic people in West Timor Indonesia; philosophy and practice in the treatment of malaria [36]	Taek et al., 2019
PubMed	Exploring antimalarial herbal plants across communities in Uganda based on electronic data [37]	Okello and Kang, 2019
	Natural products as sources of antimalarial drugs: ethnobotanical and ethnopharmacological studies [38]	Oladeji et al., 2020

Antimalarial (n = 12)		
**Database**	**Title**	**Authors, year**
Google Scholar	*In vitro* antiplasmodial investigation of medicinal plants from El Salvador [39]	Köhler et al., 2002
	In-vivo antiplasmodial activity of crude n-hexane and ethanolic extracts of *Moringa oleifera* (LAM.) seeds on *Plasmodium berghei* [40]	Olasehinde et al., 2012
	Evaluation of *Moringa oleifera* as anti-plasmodial agents in the control of malaria [41]	Ogundapo et al., 2015
	Investigating the *in-vivo* antiplasmodial properties of aqueous extract of *Moringa oleifera* Lam (Moringaceae) leaves [42]	Orman et al., 2015
	In vivo antiplasmodial activity of crude ethanolic and n-hexane extracts of *Moringa oleifera* leaves [43]	Olasehinde et al., 2016
	Evaluation of *in vivo* antimalarial activities of leaves of *Moringa oleifera* against *Plasmodium berghei* in mice [44]	Mulisa et al., 2018
	A pilot study on antimalarial effects of *Moringa oleifera* leaf extract in *Plasmodium berghei* infection in mice [45]	Nakinchat and Somsak, 2018
	Antiplasmodial activities of crude *Moringa oleifera* leaves extracts on chloroquine sensitive *Plasmodium falciparum* (3D7) [46]	Daskum et al., 2019

	Potentiation of chloroquine by antiplasmodial fraction of *Moringa oleifera* leaves in drug resistant *Plasmodium berghei* infection [47]	Ogundapo et al., 2019a
	CYP450–mediated metabolites of aqueous leaves extract of *Moringa oleifera* inhibits absorption of chloroquine in *Plasmodium yoeli yoeli* infection [48]	Ogundapo et al., 2019b
	In vivo antimalarial activities of five Ugandan medicinal plants on *Plasmodium berghei* in mice [49]	Ajayi et al., 2020b
PubMed	*Moringa oleifera* treatment increases Tbet expression in CD4+ T cells and remediates immune defects of malnutrition in *Plasmodium chabaudi*-infected mice [50]	Pilotos et al., 2020

Antimalarial and toxicity (n = 7)

**Database**	**Title**	**Authors, year**
Google Scholar	The protective effect of *Moringa oleifera* leaf extract on liver damage in mice infected with *Plasmodium berghei* ANKA [51]	Dondee et al., 2016a
	Antimalarial activities of *Moringa oleifera* leaf extract against *Plasmodium berghei* ANKA infection in ICR mice [52]	Dondee et al., 2016b
	Antiplasmodial, antipyretic, haematological and histological effects of the leaf extracts of *Moringa oleifera* in *Plasmodium berghei berghei* infected mice [53]	Olaniran et al., 2019

	Anti-plasmodial effect of *Moringa oleifera* seeds in *Plasmodium berghei* infected albino rats [54]	Obediah and Obi, 2020
	Evaluation of anti-malarial effect of *Moringa oleifera* (Lam) in *Plasmodium yoelii* infected mice [55]	Shrivastava et al., 2021
	Histopathological effects of seed oil of *Moringa oleifera* Lam. on albino mice infected with *Plasmodium berghei* (NK65) [56]	Abdulahi et al., 2022
PubMed	Antimalarial properties of aqueous crude extracts of *Gynostemma pentaphyllum* and *Moringa oleifera* leaves in combination with artesunate in *Plasmodium berghei*-Infected mice [57]	Somsak et al., 2016
	**Phytochemistry (n = 15)**	
**Database**	**Title**	**Authors, year**
Google Scholar	Effect of extraction conditions on the bioactive compounds from *Moringa oleifera* (PKM 1) seeds and their identification using LC–MS [58]	Premi and Sharma, 2017
	A comparative HPLC analysis of myricetin, quercetin and kaempferol flavonoids isolated from Gambian and Indian *Moringa oleifera* leaves [59]	Shervington et al., 2018
	Wound healing activities of *Moringa oleifera* leaves extract cultivated in Kurdistan region-Iraq [60]	Tofiq et al., 2021

ScienceDirect	Phenolic composition, antioxidant and antimicrobial activities of free and bound phenolic extracts of *Moringa oleifera* seed flour [61]	Singh et al., 2013
	Maximizing total phenolics, total flavonoids contents and antioxidant activity of *Moringa oleifera* leaf extract by the appropriate extraction method [62]	Vongsak et al., 2013
	Comparative analyses of flavonoid content in *Moringa oleifera* and *Moringa ovalifolia* with the aid of UHPLC-qTOF-MS fingerprinting [63]	Makita et al., 2016
	Bioguided extraction of phenolic compounds and UHPLC-ESI-Q-TOF-MS/MS characterization of extracts of *Moringa oleifera* leaves collected in Brazil [64]	Oldoni et al., 2019
	Extraction, purification by macrospores resin and *in vitro* antioxidant activity of flavonoids from *Moringa oliefera* leaves [65]	Hamed et al., 2019
	Ultrasonic-assisted extraction for flavonoid compounds content and antioxidant activities of India *Moringa oleifera* L. leaves: Simultaneous optimization, HPLC characterization and comparison with other methods [66]	Lin et al., 2020
	Deep eutectic solvent-based ultrasonic-assisted extraction of phenolic compounds from *Moringa oleifera* L. leaves: Optimization, comparison and antioxidant activity [67]	Wu et al., 2020

	Synergistic inhibition of isolated flavonoids from *Moringa oleifera* leaf on α-glucosidase activity [68]	Hamed et al., 2021
PubMed	*In vitro* anti-allergic activity of *Moringa oleifera* Lam. extracts and their isolated compounds [69]	Rani et al., 2019
	Impact of ultrasound extraction parameters on the antioxidant properties of *Moringa oleifera* leaves [70]	Pollini et al., 2020
	Moringa oleifera leaves: could solvent and extraction method affect phenolic composition and bioactivities? [71]	Bennour et al., 2021
	Characterization, large-scale HSCCC separation and neuroprotective effects of polyphenols from *Moringa oleifera* leaves [72]	Gao et al., 2022

References

1. Agoyi EE, Assogbadjo AE, Gouwakinnou G, Okou FA, Sinsin B. Ethnobotanical Assessment of *Moringa oleifera* Lam. in Southern Benin (West Africa). Ethnobot Res Appl. 2014 Nov;12:551-560.

2. Nda-Umar UI, Gbate M, Umar AN, Mann A. Ethnobotanical study of medicinal plants used for the treatment of malaria in Nupeland, north central Nigeria. Global J Res Med Plants Indigen Med. 2014 Apr;3:112–126.

3. Singh S, Singh R. Herbal medicinal treatment of Malaria in Aliero local government area, Kebbi, Nigeria. J Med Plants Stud. 2014 Feb;2:117-126.

4. Nondo RSO, Zofou D, Moshi MJ, Erasto P, Wanji S, Ngemenya MN, Titanji VPK, Kidukuli AW, Masimba PJ. Ethnobotanical survey and *in vitro* antiplasmodial activity of medicinal plants used to treat malaria in Kagera and Lindi regions, Tanzania. J Med Plant Res. 2015 Feb;9:179-192. doi: 10.5897/JMPR2014.5685.

5. Yaw VB, Osafo AS, Ben AG. Ethnobotanical survey of plants used in the treatment of malaria in the sekyere central district of ashanti region of Ghana. Int J Novel Res Life Sci. 2008 Nov-Dec;2:17-25.

6. Anywar G, Van't Klooster CI, Byamukama R, Willcox M, Nalumansi PA, Jong JD, Rwaburindori P, Kiremire BT. Medicinal plants used in the treatment and prevention of malaria in Cegere Sub-County, Northern Uganda. Ethnobot Res Appl. 2016 Jan;14:505-516. doi: 10.17348/era.14.0.505-516.

7. Lagnika L, Djehoue R, Yedomonhan H, Sanni A. Ethnobotanical survey of medicinal plants used in malaria management in South Benin. J Med Plant Res. 2016 Nov;10:748-756. doi: 10.5897/JMPR2016.6219.

8. Olivier TT, Francis NT, Armel S, Jackson KJ, Justin N. Ethnobotanic survey of medicinal plants used for malaria therapy in western Cameroon. J Med Plants Stud. 2016 4:248-258.

9. Dossou-Yovo HO, Vodouhè FG. Ethnobotanical survey of mangrove plant species used as medicine from ouidah to grand-popo districts, southern Benin. Am J Ethnomed. 2017 Nov;4:1-6. doi: 10.21767/2348-9502.100008.

10. Igberaese PO, Ogbole OO. Ethnobotanical survey of plants used in the treatment of typhoid and its complication(s) in Esan North East local government area, Uromi, Edo state. Niger J Pharm Res. 2018 Jan;14:175-188.

11. Mutwiwa C, Rotich B, Kauti M, Rithaa J. Ethnobotanical survey of medicinal plants in Mwala sub-county, Machakos County, Kenya. J Dis Med Plants, 2018 Aug;4:110-119. doi: 10.11648/j.jdmp.20180404.12.

12. Yaovi-Gameli A, Koffi K, Komlavi E, Amegnona A, Koffi T, Messanvi G. An Ethnobotanical survey of medicinal plants used in the preparation of “Atikédi”: Local alcoholic beverages commonly consumed in Lomé Togo. Eur Sci J. 2018 Nov;14:1-16. doi: 10.19044/esj.2018.v14n33p1.

13. Mukhtar Y, Adam A, Abdulkadir A, Yakudima I, Galalain A. Ethno botanical survey of medicinal flora used for the treatment of malaria in Madobi Town, Kano State-Nigeria. Iconic Res Eng J. 2019 Aug;3:400-409.

14. Gani AM, Kolawole OS, Dahiru M, Isyaka MS. Management of Malaria: An Account by the Indigenous People of Kashere and Its Environs, Gombe State, Nigeria. Egypt Acad J Biolog Sci. 2019 Dec;10:27-40. doi: 10.21608/EAJBSH.2019.64078.

15. Malinga GM, Baana K, Rutaro K, Opoke R, Atube F, Opika-Opoka H, Oryema C. An ethnobotanical study of plants used for the treatment of malaria in Budondo sub-county, Eastern Uganda. Ethnobot Res Appl. 2020 Jan;19:1-15. doi: 10.32859/era.19.04.1-15.

16. Ajayi CO, Elujoba AA, Kasali FM, Tenywa MG, Okella H, Weisheit A, Tolo CU, Ogwang PE. A review for selecting medicinal plants commonly used for malaria in Uganda. Afr J Pharm Pharmacol. 2020a Oct;14:347-361. doi: 10.5897/AJPP2020.5182.

17. Cole AT, Kayode J. Urban forestry and ethno medicine: The meeting point in malaria control in Ijesa Region, Nigeria. Bp Int Res Exact Sci. 2020 Jul;2:291-297. doi: 10.33258/birex.v2i3.1065.

18. Zakawa NN, Timon D, Yusuf CS, Oyebanji EO, Batta K, Jalani RT. Ethno-botanical survey and phytochemical analysis of *Moringa oleifera* in mubi local government of Adamawa state. J Med Plants Stud. 2020 Feb;8:107-111.

19. Dogara MA, Umar UU, Usman M, Sunusi N, Ladan S, Lema AA. Survey of medicinal plants used for the treatment of malaria in Kaduna state, Nigeria. Katsina J Nat Appl Sci. 2021 Sep;10:14-24.

20. Zakariya AM, Adamu A, Nuhu A, Kiri IZ. Assessment of indigenous knowledge on medicinal plants used in the management of malaria in Kafin Hausa, north-western Nigeria. Ethnobot Res Appl. 2021 Jul;22:1-18. doi: 10.32859/era.22.02.1-18.

21. Muchaia AJ, Nanvonamuquitxo SJA. Levantamento etnobotânico de plantas medicinais utilizadas pela comunidade de Nacuale, no Parque Nacional das Quirimbas, Moçambique. Nativa. 2021 Dec;9:605-611. doi: 10.31413/nativa.v9i5.12260.

22. Saikia S, Begum RA, Buragohain A. Comprehensive list of anti-malarial plants used by different communities of Assam and Arunachal Pradesh, India. Int J Mosq Res. 2021 Feb;8:63-69. doi: 10.22271/23487941.2021.v8.i2a.540.

23. Kroa E, Soumahoro A, Kouamé BY, Tiembre I, Yobouet MK. Antimalarial and antianemic medicinal plants used by traditional medicine practitioners and the populations of the Korhogo 1 health district (Poro Region, Ivory Coast). GSC Biol Pharm Sci. 2022 Apr;19:154-171. doi:10.30574/gscbps.2022.19.1.0129.

24. Tabuti JR. Herbal medicines used in the treatment of malaria in Budiope county, Uganda. J Ethnopharmacol. 2008 Feb;116:33-42. doi: 10.1016/j.jep.2007.10.036.

25. Asase A, Akwetey GA, Achel DG. Ethnopharmacological use of herbal remedies for the treatment of malaria in the Dangme West District of Ghana. J Ethnopharmacol. 2010 Jun;129:367-376. doi: 10.1016/j.jep.2010.04.001.

26. Koudouvo K, Karou DS, Kokou K, Essien K, Aklikokou K, Glitho IA, Simpore J, Sonogo R, Souza CD, Gbeassor M. An ethnobotanical study of antimalarial plants in Togo Maritime Region. J Ethnopharmacol. 2011 Mar;134:183-190. doi: 10.1016/j.jep.2010.12.011.

27. Popoola JO, Obembe OO. Local knowledge, use pattern and geographical distribution of *Moringa oleifera* Lam. (Moringaceae) in Nigeria. J Ethnopharmacol. 2013 Nov;150:682-691. doi: 10.1016/j.jep.2013.09.043.

28. Yetein MH, Houessou LG, Lougbégnon TO, Teka O, Tente B. Ethnobotanical study of medicinal plants used for the treatment of malaria in plateau of Allada, Benin (West Africa). J Ethnopharmacol. 2013 Mar;146:154-163. doi: 10.1016/j.jep.2012.12.022.

29. Ishola IO, Oreagba IA, Adeneye AA, Adirije C, Oshikoya KA, Ogunleye OO. Ethnopharmacological survey of herbal treatment of malaria in Lagos, Southwest Nigeria. J Herb Med. 2014 Dec;4:224-234. doi: 10.1016/j.hermed.2014.08.001.

30. Iyamah PC, Idu M. Ethnomedicinal survey of plants used in the treatment of malaria in Southern Nigeria. J Ethnopharmacol. 2015 Sep;173:287-302. doi: 10.1016/j.jep.2015.07.008.

31. Komlaga G, Agyare C, Dickson RA, Mensah MLK, Annan K, Loiseau PM, Champy P. Medicinal plants and finished marketed herbal products used in the treatment of malaria in the Ashanti region, Ghana. J Ethnopharmacol. 2015 Aug;172:333-346. doi: 10.1016/j.jep.2015.06.041.

32. Shah A, Rahim S. Ethnomedicinal uses of plants for the treatment of malaria in Soon Valley, Khushab, Pakistan. J Ethnopharmacol. 2017 Mar;200:84-106. doi: 10.1016/j.jep.2017.02.005.

33. Suleman S, Tufa TB, Kebebe D, Belew S, Mekonnen Y, Gashe F, Musa S, Wynendaele E, Duchateau L, Spiegeleer B. Treatment of malaria and related symptoms using traditional herbal medicine in Ethiopia. J Ethnopharmacol. 2018 Mar;213:262-279. doi: 10.1016/j.jep.2017.10.034.

34. Odoh UE, Uzor PF, Eze CL, Akunne TC, Onyegbulam CM, Osadebe PO. Medicinal plants used by the people of Nsukka Local Government Area, south-eastern Nigeria for the treatment of malaria: An ethnobotanical survey. J Ethnopharmacol. 2018 May;218:1-15. doi: 10.1016/j.jep.2018.02.034.

35. Oyeyemi IT, Akinseye KM, Adebayo SS, Oyetunji MT, Oyeyemi OT. Ethnobotanical survey of the plants used for the management of malaria in Ondo State, Nigeria. S Afr J Bot. 2019 Aug;124:391-401. doi: 10.1016/j.sajb.2019.06.003.

36. Taek MM, Banilodu L, Neonbasu G, Watu YV, EW BP, Agil M. Ethnomedicine of Tetun ethnic people in West Timor Indonesia: Philosophy and practice in the treatment of malaria. Integr Med Res. 2019 Sep;8:139-144. doi: 10.1016/j.imr.2019.05.005.

37. Okello D, Kang Y. Exploring antimalarial herbal plants across communities in Uganda based on electronic data. Evid Based Complement Altern Med. 2019 Sep;2019:3057180. doi: 10.1155/2019/3057180.

38. Oladeji OS, Oluyori AP, Bankole DT, Afolabi TY. Natural products as sources of antimalarial drugs: ethnobotanical and ethnopharmacological studies. Scientifica. 2020 May;2020:7076139. doi: 10.1155/2020/7076139.

39. Köhler I, Jenett-Siems K, Siems K, Hernández MA, Ibarra RA, Berendsohn WG, Bienzle U, Eich E. *In vitro* antiplasmodial investigation of medicinal plants from El Salvador. Z Naturforsch C. 2002 Jan;57:277-281. doi: 10.1515/znc-2002-3-413.

40. Olasehinde GI, Ayanda OI, Ajayi AA, Nwabueze AP. *In-vivo* antiplasmodial activity of crude n-hexane and ethanolic extracts of *Moringa oleifera* (Lam.) seeds on *Plasmodium berghei*. Int J Med Plant Res. 2012 Oct;1:050-054.

41. Ogundapo SS, Ezeanyika LUS, Uzoegwu PN, Soniran OT, Okoro DO, Okoronkwo I, Okoro JA, Okochi PC, Chukwunwike OO. Evaluation of *Moringa oleifera* as anti-plasmodial agents in the control of malaria. Niger J Parasitol. 2015 Mar;36:22-27.

42. Orman E, Addo P, Ofori MF, Reimmel KA. Investigating the *in-vivo* antiplasmodial properties of aqueous extract of *Moringa oleifera* Lam (Moringaceae) leaves. Br J Pharm Res. 2015 Feb;5:419-430. doi: 10.9734/BJPR/2015/15714.

43. Olasehinde GI, Ayanda OI, Egwari LO, Ajayi AA, Awofeso T. *In vivo* antiplasmodial activity of crude ethanolic and N-hexane extracts of *Moringa oleifera* leaves. Int J Agri Bio. 2016 18:906-910. doi: 10.17957/IJAB/15.0161.

44. Mulisa E, Girma B, Tesema S, Yohannes M, Zemene E, Amelo W. Evaluation of *in vivo* antimalarial activities of leaves of *Moringa oleifera* against *Plasmodium berghei* in mice. Jundishapur J Nat Pharm Prod. 2018 Feb;13:e60426. doi: 10.5812/jjnpp.60426.

45. Nakinchat S, Somsak V. A pilot study on antimalarial effects of *Moringa oleifera* leaf extract in *Plasmodium berghei* infection in mice. Walailak J Sci Tech. 2017 Nov;15:151-156. doi: 10.48048/wjst.2018.4593.

46. Daskum AM, Godly C, Qadeer MA. Antiplasmodial activities of crude *Moringa oleifera* leaves extracts on chloroquine sensitive *Plasmodium falciparum* (3D7). Bayero J Pure Appl Sci. 2019 Nov;12:315-320. doi: 10.4314/bajopas.v12i1.48S.

47. Ogundapo SS, Ezeanyika LU, Uzoegwu PN, Soniran OT. Potentiation of chloroquine by antiplasmodial fraction of *Moringa oleifera* leaves in drug resistant *Plasmodium berghei* infection. Invest Med Chem Pharmacol. 2019a 2:22. doi: 10.31183/imcp.2019.00022.

48. Ogundapo SS, Soniran OT, Suleiman JB, Chigozie K, Ngobidi NAO, Olugbue VU, Chukwunwike OO, Olatunji ID. CYP450–mediated metabolites of aqueous leaves extract of *Moringa oleifera* inhibits absorption of chloroquine in *Plasmodium yoeli yoeli* infection. Invest Med Chem Pharmacol. 2019b 2:23. doi: 10.31183/imcp.2019.00023.

49. Ajayi CO, Elujoba AA, Okella H, Oloro J, Raymond A, Weisheit A, Tolo CU, Ogwang PE. *In vivo* antimalarial activities of five Ugandan medicinal plants on *Plasmodium berghei* in mice. European J Med Plants. 2020b Aug;31:1-13. doi: 10.9734/ejmp/2020/v31i1230300.

50. Pilotos J, Ibrahim KA, Mowa CN, Opata MM. *Moringa oleifera* treatment increases Tbet expression in CD4+ T cells and remediates immune defects of malnutrition in *Plasmodium chabaudi*-infected mice. Malar J. 2020 Feb;19:62. doi: 10.1186/s12936-020-3129-8.

51. Dondee K, Borkaew P, Klubsri C, Bootprom P, Saiphet B, Somsak V. The protective effect of *Moringa oleifera* leaf extract on liver damage in mice infected with *Plasmodium berghei* ANKA. J Coast Life Med. 2016a Sep;4:742-746. doi: 10.12980/jclm.4.2016J6-150.

52. Dondee K, Bootprom P, Saiphet B, Borkaew P, Klubsri C, Somsak V. Antimalarial activities of *Moringa oleifera* leaf extract against *Plasmodium berghei* ANKA infection in ICR mice. Int J Innov Res Med Sci. 2016b Jul;1:194-201.

53. Olaniran O, Adetuyi FC, Omoya FO, Odediran SA, Hassan-olajokun RE, Awoyeni EA, Odetoyin BW, Adesina A, Awe A, Bejide RA, Odujoko O, Akinyemi LO, Oyetoke OO, Afolayan DO. Antiplasmodial, antipyretic, haematological and histological effects of the leaf extracts of *Moringa oleifera* in *Plasmodium berghei berghei* infected mice. J Adv Med Med Res. 2019 Apr;29:1-13. doi: 10.9734/JAMMR/2019/v29i430083.

54. Obediah GA, Obi NC. Anti-plasmodial effect of *Moringa oleifera* seeds in *Plasmodium berghei* infected albino rats. Biochem Pharmacol. 2020 Jan;9:2167-0501. doi: 10.35248/2167-0501.20.9.268.

55. Shrivastava M, Prasad A, Kumar D. Evaluation of anti malarial effect *Moringa oleifera* (Lam) in *Plasmodium yoelii* infected mice. Indian J Pharm Sci. 2021 Sep;83:1221-1228. doi: 10.36468/pharmaceutical-sciences.877.

56. Abdulahi SK, Dada EO, Adebayo RO. Histopathological effects of seed oil of *Moringa oleifera* Lam. on albino mice infected *with Plasmodium berghei* (NK65). Adv J Grad Res. 2022 Dec;11:71-79. doi: 10.21467/ajgr.11.1.71-79.

57. Somsak V, Borkaew P, Klubsri C, Dondee K, Bootprom P, Saiphet B. Antimalarial properties of aqueous crude extracts of *Gynostemma pentaphyllum* and *Moringa oleifera* leaves in combination with artesunate in *Plasmodium berghei*-infected mice. J Trop Med. 2016 Oct;2016:8031392. doi: 10.1155/2016/8031392.

58. Premi M, Sharma HK. Effect of extraction conditions on the bioactive compounds from *Moringa oleifera* (PKM 1) seeds and their identification using LC–MS. J Food Meas Charact. 2017 Mar;11:213-225. doi: 10.1007/s11694-016-9388-y.

59. Shervington LA, Li BS, Shervington AA, Alpan N, Patel R, Muttakin U, Mulla E. A comparative HPLC analysis of myricetin, quercetin and kaempferol flavonoids isolated from Gambian and Indian *Moringa oleifera* leaves. Int J Chem. 2018 Nov;10:28-37. doi: 10.5539/ijc.v10n4p28.

60. Tofiq SA, Azeez HA, Othman HH. Wound healing activities of *Moringa oleifera* leaves extract cultivated in Kurdistan region-Iraq. Jordan J Biol Sci. 2021 Dec;14:637-645. doi: 10.54319/jjbs/140403.

61. Singh RG, Negi PS, Radha C. Phenolic composition, antioxidant and antimicrobial activities of free and bound phenolic extracts of *Moringa oleifera* seed flour. J Funct Foods. 2013 Oct;5:1883-1891. doi: 10.1016/j.jff.2013.09.009.

62. Vongsak B, Sithisarn P, Mangmool S, Thongpraditchote S, Wongkrajang Y, Gritsanapan W. Maximizing total phenolics, total flavonoids contents and antioxidant activity of *Moringa oleifera* leaf extract by the appropriate extraction method. Ind Crops Prod. 2013 Jan;44:566-571. doi: 10.1016/j.indcrop.2012.09.021.

63. Makita C, Chimuka L, Steenkamp P, Cukrowska E, Madala E. Comparative analyses of flavonoid content in *Moringa oleifera* and *Moringa ovalifolia* with the aid of UHPLC-qTOF-MS fingerprinting. S Afr J Bot. 2016 Jul;105:116-122. doi: 10.1016/j.sajb.2015.12.007.

64. Oldoni TLC, Merlin N, Karling M, Carpes ST, Alencar SM, Morales RGF, Silva EA, Pilau EJ. Bioguided extraction of phenolic compounds and UHPLC-ESI-Q-TOF-MS/MS characterization of extracts of *Moringa oleifera* leaves collected in Brazil. Food Res Int. 2019 Nov;125:108647. doi: 10.1016/j.foodres.2019.108647.

65. Hamed YS, Abdin M, Akhtar HMS, Chen D, Wan P, Chen G, Zeng X. Extraction, purification by macrospores resin and *in vitro* antioxidant activity of flavonoids from *Moringa oliefera* leaves. S Afr J Bot. 2019 Aug;124:270-279. doi: 10.1016/j.sajb.2019.05.006.

66. Lin X, Wu L, Wang X, Yao L, Wang L. Ultrasonic-assisted extraction for flavonoid compounds content and antioxidant activities of India *Moringa oleifera* L. leaves: Simultaneous optimization, HPLC characterization and comparison with other methods. J Appl Res Med Aromat Plants. 2021 Feb;20:100284. doi: 10.1016/j.jarmap.2020.100284.

67. Wu L, Li L, Chen S, Wang L, Lin X. Deep eutectic solvent-based ultrasonic-assisted extraction of phenolic compounds from *Moringa oleifera* L. leaves: Optimization, comparison and antioxidant activity. Sep Purif Technol. 2020 Sep;247:117014. doi: 10.1016/j.seppur.2020.117014.

68. Hamed YS, Abdin M, Rayan AM, Akhtar HMS, Zeng X. Synergistic inhibition of isolated flavonoids from *Moringa oleifera* leaf on α-glucosidase activity. LWT. 2021 Apr;141:111081. doi: 10.1016/j.lwt.2021.111081.

69. Rani NZA, Kumolosasi E, Jasamai M, Jamal JA, Lam KW, Husain K. *In vitro* anti-allergic activity of *Moringa oleifera* Lam. extracts and their isolated compounds. BMC Complement Altern Med. 2019 Dec;19:361. doi: 10.1186/s12906-019-2776-1.

70. Pollini L, Tringaniello C, Ianni F, Blasi F, Manes J, Cossignani L. Impact of ultrasound extraction parameters on the antioxidant properties of *Moringa oleifera* leaves. Antioxidants. 2020 Mar;9:277. doi: 10.3390/antiox9040277.

71. Bennour N, Mighri H, Bouhamda T, Mabrouk M, Apohan E, Yesilada O, Küçükbay H, Akrout A. *Moringa oleifera* leaves: could solvent and extraction method affect phenolic composition and bioactivities?. Prep Biochem Biotechnol. 2021 Mar;51:1018-1025. doi: 10.1080/10826068.2021.1891550.

72. Gao Q, Wei Z, Liu Y, Wang F, Zhang S, Serrano C, Li L, Sun B. Characterization, Large-Scale HSCCC Separation and Neuroprotective Effects of Polyphenols from *Moringa oleifera* Leaves. Molecules. 2022 Jan;27:678. doi: 10.3390/molecules27030678.
